# WWOX Induction Promotes Bcl-X_L_ and Mcl-1 Degradation Through a Lysosomal Pathway upon Stress Responses

**DOI:** 10.3390/cells15030270

**Published:** 2026-01-31

**Authors:** Yu-Han Su, Wei Chiang, Yi-Yu Wang, Yi-Hsi Kung, Pai-Shan Cheng, Tsung-Hao Chang, Nan-Shan Chang, Feng-Jie Lai, Li-Jin Hsu

**Affiliations:** 1 Department of Medical Laboratory Science and Biotechnology, College of Medicine, National Cheng Kung University, Tainan 701401, Taiwan; 2 Institute of Basic Medical Sciences, College of Medicine, National Cheng Kung University, Tainan 701401, Taiwan; 3 Department of Dermatology, Chi Mei Medical Center, Tainan 710402, Taiwan; 4 Graduate Institute of Biomedical Sciences, China Medical University, Taichung 404328, Taiwan; 5 Center for General Education, Southern Taiwan University of Science and Technology, Tainan 710301, Taiwan; 6 Center of Infectious Disease and Signaling Research, College of Medicine, National Cheng Kung University, Tainan 701401, Taiwan; 7 Research Center for Medical Laboratory Biotechnology, College of Medicine, National Cheng Kung University, Tainan 701401, Taiwan

**Keywords:** tumor suppressor, lysosome, protein degradation, oxidative stress, Bcl-2 family

## Abstract

The human *WWOX* gene resides on a common fragile site and is frequently deleted or altered during DNA replication. *WWOX* mutations are associated with various human diseases, including cancer, neurodegeneration, and developmental deficits. However, the regulation of WWOX expression remains largely unclear. We demonstrated that stress responses, including serum deprivation, oxidative stress, and anticancer drug treatment, increase WWOX expression in human SCC-15 cells and wild-type mouse embryonic fibroblasts (MEFs) through transcriptional activation. Serum deprivation induces higher levels of reactive oxygen species and cell death in *Wwox*^+/+^ than *Wwox*^−/−^ MEFs. Anti-apoptotic Bcl-2 family proteins regulate mitochondrial homeostasis and prevent serum deprivation-induced oxidative stress and cell death. Our results showed that serum starvation decreases protein expression levels of Bcl-X_L_ and Mcl-1 in *Wwox*^+/+^ but not in *Wwox*^−/−^ MEFs. Serum starvation also fails to downregulate Bcl-X_L_ and Mcl-1 protein expression in WWOX-knockdown SCC-15 cells. Replenishment of ectopic WWOX induces downregulation of Bcl-X_L_ and Mcl-1 protein levels in *Wwox*^−/−^ MEFs after serum starvation. We determined that WWOX-mediated downregulation of Bcl-XL and Mcl-1 is accomplished through a lysosome-dependent protein degradation pathway. Moreover, a decline in reactive oxygen species generation by pretreatment of *Wwox*^+/+^ MEFs with an antioxidant N-acetyl-L-cysteine leads to decreased WWOX induction upon serum starvation. Taken together, our results suggest that stress stimuli trigger WWOX induction by elevating the production of reactive oxygen species in cells, which promotes the degradation of Bcl-XL and Mcl-1 proteins via a lysosome-mediated pathway, thereby further aggravating oxidative stress and cell death.

## 1. Introduction

*The WWOX* gene resides within a common fragile site, *FRA16D,* on human chromosome 16q23.3-24.1 [1,2]. The phenotypes of pediatric patients with homozygous loss-of-function mutation of the *WWOX* gene include microcephaly, cerebellar ataxia associated with epileptic seizures and intellectual disability, retinopathy, profound developmental delay, and early postnatal lethality [3,4,5,6,7,8,9]. The neurodevelopmental and neurodegenerative deficits in *Wwox* knockout mice clearly recapitulate the key features of neuropathies in patients with WWOX-related epileptic encephalopathy (WOREE) syndrome [10]. Significantly decreased expression of WWOX protein has been examined in the hippocampal neurons of patients with Alzheimer’s disease [11]. Suppression of WWOX protein expression by small interfering RNA (siRNA) induces Tau hyperphosphorylation, protein aggregation, and neurofibrillary tangle formation in neuroblastoma SK-N-SH cells, suggesting a crucial role of WWOX in blocking Tau phosphorylation in the degenerative neurons of Alzheimer’s disease [11]. WWOX has been shown to promote neuronal differentiation via suppressing GSK-3β activity [10]. WWOX also inhibits HIF-1α and RUNX2 for regulating glucose and bone metabolism, respectively [12,13]. Therefore, delineating the regulation of WWOX expression and function in cells may help achieve a comprehensive understanding of the pathophysiological changes due to WWOX deficiency in patients.

WWOX has been found to exert tumor suppressor properties in cancer development and progression. Frequent deletion, translocation, and mutations of the fragile *WWOX* gene have been detected in human cancer specimens, including breast, ovarian, prostate, lung, liver, bladder, esophageal, and pancreatic cancers [2]. Importantly, WWOX possesses proapoptotic functions through interacting with transcription factors AP-2γ, c-Jun, p73, and ΔNp63α to block their nuclear translocation and induction of prosurvival genes, thereby suppressing cancer cell growth [14,15,16,17,18]. WWOX also binds to p53 for sensitizing the cells to apoptosis induced by tumor necrosis factor, ultraviolet light, and chemotherapeutic drugs [19,20]. Moreover, WWOX has the capability to regulate DNA damage response (DDR). WWOX interacts with Brca1 to promote non-homologous end-joining repair of double-strand DNA breaks [21]. WWOX also enhances ATM and ATR kinase activities for facilitating DNA repair [22,23]. It is likely that WWOX upregulation in the hyperplastic cells during the early stage of cancer development contributes to counteracting the accumulation of deleterious mutations in these cells for maintaining genome integrity [24]. If DNA lesions are beyond the repair capacity, the DDR machinery may trigger apoptosis or senescence to prevent propagation of damaged DNA [25]. Loss of WWOX expression may lead to senescence escape, genome instability, and cancer deterioration [26].

Cancer cells usually demonstrate remarkable endurance under various kinds of stress, adapting and even thriving in challenging tumor microenvironments. However, how cells respond to environmental stress through regulating the expression of WWOX is not clear. In this study, we determined significantly increased *WWOX* gene transcription and protein expression in cells in response to stress. WWOX upregulation decreased the amounts of prosurvival Bcl-X_L_ and Mcl-1 through a lysosome-dependent protein degradation pathway. The resulting accumulation of reactive oxygen species (ROS) caused augmentation of stress-elicited responses in cells, suggesting the crucial effect of WWOX induction in regulating Bcl-X_L_ and Mcl-1 protein degradation and ROS generation.

## 2. Materials and Methods

### 2.1. Cells and Chemicals

Human squamous cell carcinoma (SCC)-15 cells from American Type Culture Collection (ATCC; Manassas, VA, USA) were cultured in a 1:1 mixture of Dulbecco’s modified Eagle’s medium (DMEM) and a nutrient mixture F12 (Invitrogen, Carlsbad, CA, USA) supplemented with 2 mM L-glutamine, 0.5 mM sodium pyruvate, 400 ng/mL hydrocortisone (Sigma, St. Louis, MO, USA) and 10% fetal bovine serum (FBS). HeLa Tet-On cells that stably express reverse tetracycline-controlled transactivator protein (Takara Bio USA, Inc., Kusatsu, Japan) were cultured in DMEM supplemented with 100 μg/mL G418 (Calbiochem, Darmstadt, Germany) and 10% FBS. Wild-type and homozygous *Wwox* knockout mouse embryonic fibroblast (MEF) lines were generated as previously described [26] and maintained in RPMI-1640 medium containing 10% FBS. All cells were cultured at 37 °C in 5% CO_2_. Cell viability was detected using a Dojindo cell counting kit-8 (Kumamoto, Japan) according to the manufacturer’s instructions.

Etoposide, cisplatin, hydrogen peroxide, doxycycline hyclate, chloroquine diphosphate salt, (2S,3S)-*trans*-epoxysuccinyl-L-leucylamido-3-methylbutane ethyl ester (E64d), MG132, cycloheximide, and N-acetyl-L-cysteine (NAC) were purchased from Sigma (St. Louis, MO, USA). Pepstatin A was obtained from Tocris (Bristol, UK).

### 2.2. Western Blotting

Cells were scraped off from the dishes at the indicated time points and collected into a 15 mL centrifuge tube. After centrifugation at 2000 rpm for 10 min at 4 °C, the supernatant was discarded. The cells were lysed with an appropriate volume of cold lysis buffer containing 1% Nonidet P40, 0.1% sodium dodecyl sulfate (SDS), 0.5% Tween 20, 10 mM NaF, 10 mM Na_4_P_2_O_7_·10H_2_O, 10 mM Na_3_VO_4,_ and a 1:20 dilution of a protease inhibitor cocktail (Sigma) in PBS by pipetting. The cell lysates were transferred into a microcentrifuge tube and incubated on ice for 15 min. After centrifugation at 13,000 rpm for 15 min at 4 °C, the supernatant was collected into a new tube. The protein content was determined using a protein assay dye reagent (Bio-Rad, Hercules, CA, USA) and adjusted using the lysis buffer and mixed with 5× sample buffer (250 mM Tris-HCl (pH 6.8), 500 mM dithiothreitol, 10% SDS, 0.1% bromophenol blue, and 50% glycerol). The samples were then heated for 10 min at 95 °C. Equal amounts of cellular proteins were analyzed by SDS-polyacrylamide gel electrophoresis and transferred to PVDF membranes. The antibodies used in the detection of specific proteins were anti-Bcl-X_L_, anti-Mcl-1, anti-Bcl-2, anti-Bax, anti-Bak, anti-Bad (Cell Signaling, Danvers, MA, USA), anti-green fluorescence protein (GFP), anti-WWOX (GeneTex), and anti-β-actin (Sigma). The secondary antibodies used were horseradish peroxidase-conjugated goat anti-rabbit IgG or horse anti-mouse IgG (Cell Signaling). Enhanced chemiluminescence (ECL, Amersham, UK) was detected as described [20]. Quantitative densitometry of the immunoblots was analyzed using the ImageJ program (Version 1.54p; National Institutes of Health, Bethesda). The relative amount of protein expression in cells was calculated by normalizing the densitometric value obtained from each protein with that from β-actin, and the results were depicted by the ratio of each sample to the untreated control/wild-type group.

### 2.3. RNA Extraction, Reverse Transcription (RT), and Real-Time Polymerase Chain Reaction (PCR)

Cells were scraped off and collected into a 15 mL centrifuge tube. After centrifugation at 2000 rpm for 10 min at 4 °C, the supernatant was removed, and the cells were resuspended using 1 mL TRIzol reagent. The cell lysate was transferred into a microcentrifuge tube. The sample was mixed with 200 μL chloroform vigorously for 15 s and then incubated at room temperature for 5 min. After centrifugation at 13,000 rpm for 15 min at 4 °C, the colorless aqueous phase at the upper layer containing RNA was collected carefully into a new tube and mixed with an equivalent volume of isopropanol. The tubes were inverted gently 2~3 times, and the mixture was incubated on ice for 10 min. After centrifugation at 13,000 rpm for 10 min at 4 °C, the supernatant was removed. The RNA pellet was washed with 1 mL 75% ethanol by inverting the tube several times. After centrifugation at 13,000 rpm for 10 min at 4 °C, the supernatant was removed, and the RNA pellet was air-dried at room temperature. The RNA samples were dissolved in 20 μL H_2_O, and the concentration of RNA was measured using a spectrophotometer. RNA samples were used for generating cDNA using a reverse transcription kit (Promega, Woods, WI, USA).

Quantitative real-time PCR analysis was conducted in triplicate using SYBR Green detection methodology on a StepOnePlus™ Real-Time PCR System (Applied Biosystems, Waltham, MA, USA) as described previously [20]. Each reaction was carried out in a total volume of 20 μL containing 10 μL of SYBR Green master mix, 0.3 μM of each primer, and 1 μL of cDNA sample. The primer pairs used in this study are listed in Table 1. The thermal cycling conditions were set as follows: initial denaturation at 95 °C for 10 min, followed by 40 cycles of denaturation at 95 °C for 15 s, annealing at 60 °C for 30 s, and extension at 72 °C for 30 s. Data were analyzed using a comparative *Ct* method, where Δ*Ct* = *Ct* (gene of interest) − *Ct* (β-actin). The relative level of mRNA expression was calculated by normalizing the Δ*Ct* obtained from each sample with that from the untreated cells. For verification, the PCR products were used for electrophoresis on 2% agarose gels run in TAE buffer, visualized by ethidium bromide staining, and analyzed using an ImageQuant 300 imaging system (GE Healthcare Life Sciences, Marlborough, MA, USA) as described [20].

### 2.4. Transfection and Lentiviral shRNA-Mediated Knockdown

Generation of the constructs expressing doxycycline-induced WWOX was described previously [26]. Cells were transfected with the expression construct using a BioRad Gene Pulser System (200 V and 50 mSec, square wave). After electroporation, cells were cultured in complete medium supplemented with FBS in 10 cm dishes for 24 h and used for experiments. For gene silencing via RNA interference, cells in 6 cm dishes were infected with lentivirus to deliver short hairpin RNA (shRNA). The shRNA sequences used in this study are listed in Table 1. The infected cells were incubated in complete medium supplemented with FBS at 37 °C in 5% CO_2_ for at least 24 h and then selected in the presence of 1 μg/mL puromycin.

### 2.5. Flow Cytometry

A cationic fluorescent dye, rhodamine 123 (Sigma), was used to monitor the changes in mitochondrial membrane potential in living cells. In brief, the cells were resuspended and incubated with 5 μM rhodamine 123 in serum-free medium at 37 °C in the dark for 30 min. After centrifugation at 2000 rpm for 10 min, the supernatant was discarded. The cells were washed twice with serum-free medium and analyzed by flow cytometry. For cell cycle analysis, cells were treated with 0.25% trypsin-EDTA at room temperature for 3–5 min until detachment occurred. The cell suspension was transferred into a centrifuge tube. After centrifugation at 2000 rpm for 5 min at 4 °C, the supernatant was discarded, and the tube was tapped to resuspend the cells. The cells were fixed with 2 mL 70% ethanol/PBS and then incubated for 30 min at 4 °C. After centrifugation at 2000 rpm for 10 min at 4 °C, the supernatant was removed carefully, and the cells were resuspended by tapping the tube. The cells were then stained with a propidium iodide staining solution containing 40 μg/mL propidium iodide (Sigma) and 100 μg/mL RNase A (Invitrogen, Carlsbad, CA, USA) in PBS in the dark for 30 min at room temperature. The cell cycle distribution was analyzed using a FACScan (Becton Dickinson, Mountain View, CA, USA) with excitation wavelength set at 488 nm.

### 2.6. Statistical Analysis

Data were presented as means ± standard deviation (s.d.). Statistical significance was determined using Student’s *t*-test. The differences were considered significant as *p-values* were less than 0.05.

## 3. Results

### 3.1. Stress Stimulation Increases WWOX Expression in Human Cancer Cells

**Figure 1:**
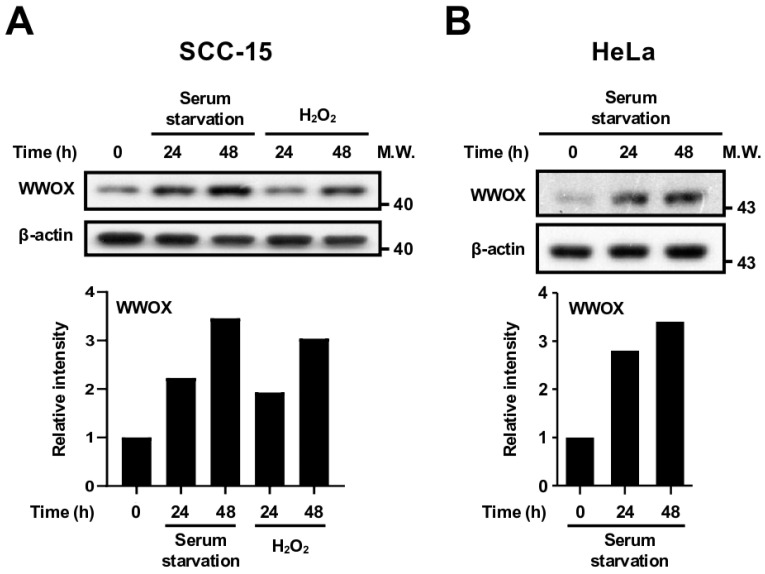
Stress stimulation upregulates WWOX protein expression in human SCC-15 and HeLa cells. (**A**) SCC-15 cells were treated with serum-free medium or 100 μM H_2_O_2_ for 24 and 48 h. Total cellular protein extracts were prepared, and WWOX protein levels in cells were determined by Western blot analysis. (**B**) HeLa cells were treated with serum-free medium for 24 and 48 h. WWOX protein expression levels in cells were determined by Western blot analysis. β-actin was used as an internal control. The lower panel shows densitometric analysis. The representative results from at least four repeated experiments are shown. M.W., molecular weight (kDa).

Under stress conditions, WWOX is activated via phosphorylation at tyrosine 33 by tyrosine kinase Src and physically interacts with p53, p73, ΔNp63α, and c-Jun for modulating cancer cytotoxicity [14,16,17]. However, whether stress stimulation regulates the expression of WWOX is largely unclear. To understand how cells react to stress responses, we first tested various stress stimuli that cancer cells may encounter in the tumor microenvironment, such as serum starvation, oxidative stress, and chemotherapeutic drugs. We determined that the expression levels of WWOX protein were markedly increased in human SCC-15 (Figure 1A) and cervical carcinoma HeLa cells (Figure 1B) after serum deprivation in a time-dependent manner. Moreover, oxidative stress is induced by treatment of SCC-15 cells with 100 μM hydrogen peroxide (H_2_O_2_), which also upregulated WWOX protein expression (Figure 1A). Etoposide is a topoisomerase inhibitor that may generate DNA breaks in cells. Cisplatin causes DNA crosslinking and interferes with DNA replication. Both etoposide and cisplatin induce replication stress, cell cycle arrest, and eventually apoptosis in fast proliferating cells. Our data revealed that treatment of SCC-15 cells with anticancer drugs etoposide and cisplatin also increased the expression of WWOX protein (Figure S1).

**Figure 2:**
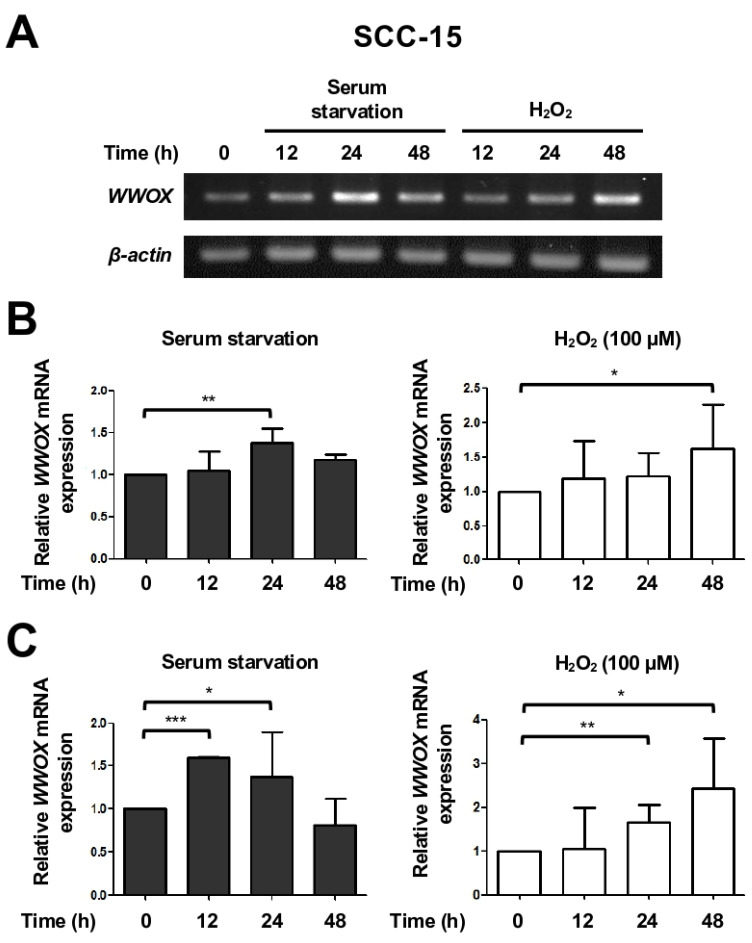
Stress induces *WWOX* gene transcription in SCC-15 cells. (**A**,**B**) SCC-15 cells were treated with serum-free medium or 100 μM H_2_O_2_ for 12, 24 and 48 h. The RNA samples were extracted, and *WWOX* mRNA levels in cells were determined by reverse transcription PCR, followed by agarose gel electrophoresis (**A**) and densitometric analysis (**B**). (**C**) Quantitative real-time PCR was performed to detect *WWOX* mRNA expression levels in SCC-15 cells treated with serum-free medium or 100 μM H_2_O_2_ for the indicated time periods. β-actin was used as an internal control. The relative *WWOX* mRNA expression levels were analyzed by normalizing the data obtained from each sample (n = 3) with the results from untreated cells. *, *p* ≤ 0.05; **, *p* ≤ 0.01; ***, *p* ≤ 0.005.

To further investigate whether the upregulation of WWOX protein expression is due to gene activation, we checked *WWOX* mRNA levels by RT-PCR. Serum starvation for 24 h indeed increased *WWOX* mRNA levels in SCC-15 cells (Figure 2A,B; *left*), although downregulation of *WWOX* mRNA expression due to a possible negative feedback mechanism after 48 h following serum starvation was detected. Oxidative stress induced by H_2_O_2_ treatment also increased *WWOX* gene transcription in SCC-15 cells (Figure 2A,B; *right*). We ascertained the induction of *WWOX* gene expression by performing quantitative real-time PCR for measuring the amounts of *WWOX* mRNA in the treated SCC-15 cells (Figure 2C). Together, we determined that various stress stimuli activate *WWOX* gene transcription and increase WWOX protein levels in human cancer cells.

### 3.2. Serum Growth Factor Deprivation and Oxidative Stress Upregulate Wwox mRNA and Protein Expression in Primary Mouse Fibroblasts

**Figure 3:**
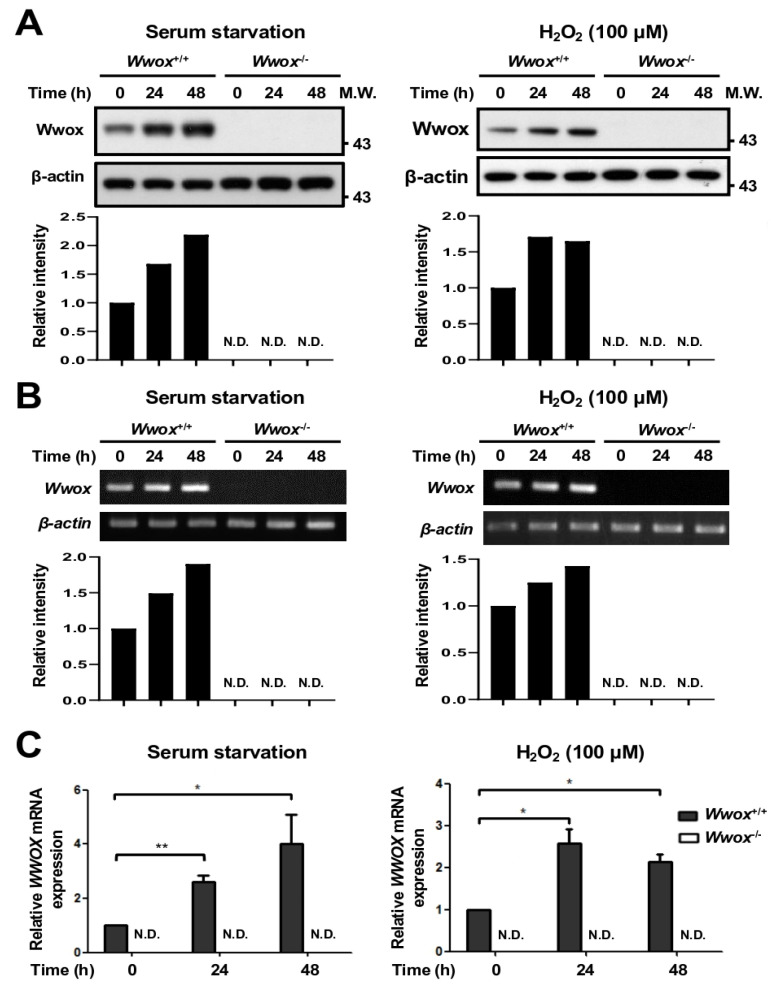
Serum starvation and oxidative stress induce WWOX upregulation in primary wild-type MEFs. (**A**) *Wwox*^+/+^ and *Wwox*^−/−^ MEFs were treated with serum-free RPMI-1640 medium or 100 μM H_2_O_2_ for 24 and 48 h. Following stimulation, WWOX protein upregulation was observed in *Wwox*^+/+^ MEFs by Western blot analysis. No expression signals were detected in *Wwox*^−/−^ MEFs using a specific anti-WWOX antibody, confirming the knockout efficiency of *Wwox* in mice. β-actin was used as an internal control. The representative results from at least four repeated experiments are shown. (**B**,**C**) RNA samples were collected from *Wwox*^+/+^ and *Wwox*^−/−^ MEFs treated with serum-free RPMI-1640 medium or 100 μM H_2_O_2_ for 24 and 48 h. *WWOX* mRNA levels in cells were determined by reverse transcription PCR and agarose gel electrophoresis (**B**). (**C**) Quantitative real-time PCR was performed to detect relative levels of *WWOX* mRNA expression in MEFs (n = 3). The expression of *Wwox* mRNA in *Wwox*^-/-^ MEFs was not detectable (N.D.). *, *p* ≤ 0.05; **, *p* ≤ 0.01.

To analyze whether primary cells respond to stress stimulation with upregulation of WWOX protein expression, MEFs were tested in this study. As shown in Figure 3A, serum starvation and H_2_O_2_ treatment induced Wwox protein upregulation in wild-type (*Wwox*^+/+^) MEFs in a time-dependent manner. The absent expression of 46-kDa Wwox protein in fibroblasts isolated from *Wwox* homozygous knockout (*Wwox*^−/−^) mouse fetuses at E14.5 was determined (Figure 3A) [26], and the specificity of the polyclonal antibody generated using an N-terminal segment of WWOX was affirmed [11]. As expected, serum starvation and H_2_O_2_ treatment increased the mRNA expression levels of *Wwox* in wild-type MEFs (Figure 3B,C), suggesting that stress stimulation may induce *Wwox* upregulation via transcriptional activation in primary murine fibroblasts. We repeated each experiment at least four times and obtained consistent results.

### 3.3. Wwox-Deficient MEFs Are More Resistant to Starvation-Induced Cell Death

**Figure 4:**
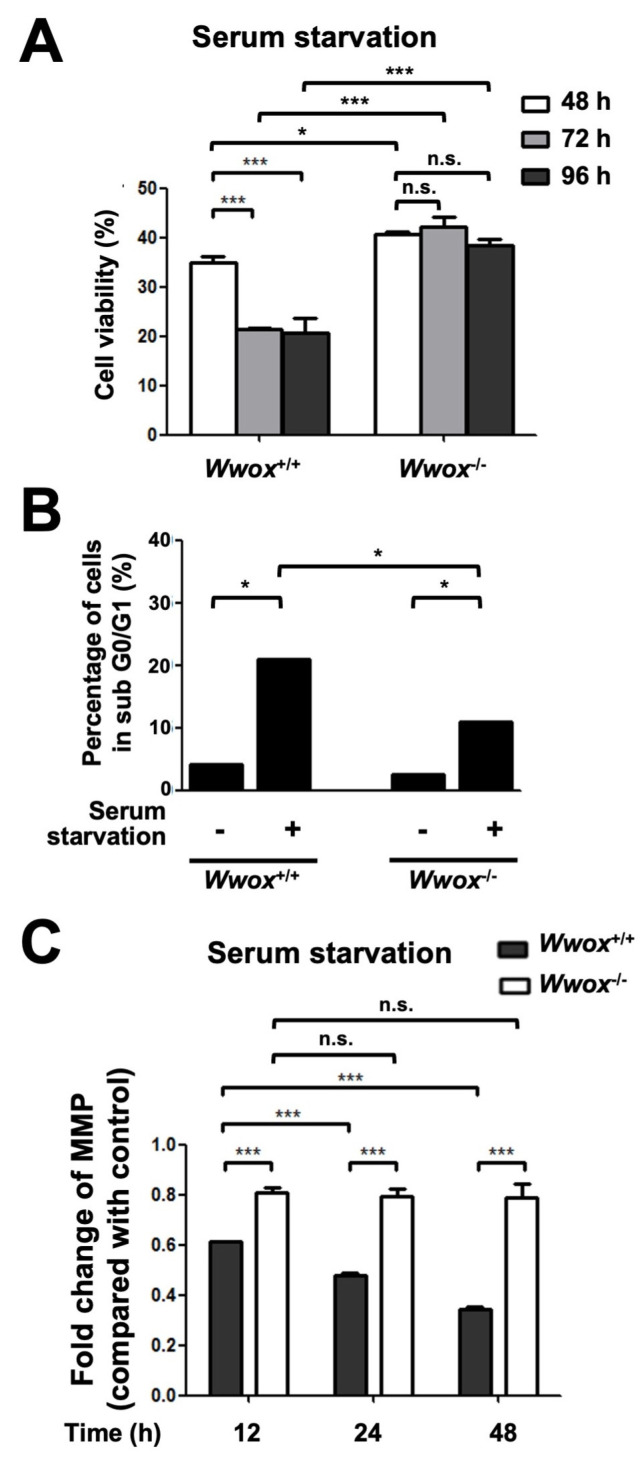
A higher level of cell death induced by long-term starvation occurs in wild-type MEFs than in *Wwox*-deficient MEFs. (**A**) *Wwox*^+/+^ and *Wwox*^−/−^ MEFs were treated with serum-free medium for 48, 72, and 96 h. Cell viability was analyzed by a WST-8 cell counting kit. The percentages of cell viability (n = 4) were normalized with control cells cultured with medium containing 10% serum. (**B**) *Wwox*^+/+^ and *Wwox*^−/−^ MEFs were treated with serum-free medium for 72 h. The percentages of cells in subG0/G1 were analyzed by propidium iodide staining and flow cytometry (n = 3). (**C**) Significant reduction in MMP in *Wwox*^+/+^ MEFs was detected by staining the cells with rhodamine 123 after serum starvation for 12, 24, and 48 h. MMP in *Wwox*^−/−^ MEFs remained relatively stable following serum starvation (n = 4). *, *p* ≤ 0.05; ***, *p* ≤ 0.005; n.s., not significant.

WWOX is crucial for tumor necrosis factor-, ultraviolet light-, staurosporine-, and p53-induced cell death [24]. We have demonstrated that *Wwox* deficiency led to senescence escape and accelerated cell proliferation in MEFs [26]. To study whether WWOX increases the induction of cell death upon long-term serum growth factor deprivation, we analyzed the viability of MEFs following serum starvation using a WST-8 assay kit (Figure 4A). Although decreased cell viabilities were detected in both *Wwox*^+/+^ and *Wwox*^−/−^ MEFs after serum starvation, *Wwox*^+/+^ MEFs showed a significantly enhanced reduction in cell viability as compared with *Wwox*^−/−^ MEFs after serum starvation for 72 h (Figure 4A). To further test whether serum starvation-induced *Wwox* upregulation promotes apoptosis in MEFs, we measured the extent of cell apoptosis by propidium iodide staining followed by flow cytometric analysis (Figure 4B). Our data revealed that serum starvation for 48 h induced a higher percentage of cells in sub G0/G1 phase in *Wwox*^+/+^ than in *Wwox*^−/−^ MEFs (Figure 4B). These results suggest that serum starvation-induced *Wwox* upregulation is associated with the induction of cell apoptosis. A sequence targeting mitochondria has been identified within the SDR domain of WWOX protein [19]. Under stress conditions, WWOX is activated via phosphorylation at tyrosine 33 by Src protein kinase and translocates to the mitochondria [14,27,28]. However, the functional relevance of WWOX translocation to mitochondria is still unclear. The electron transport chain creates an electrochemical gradient for generating mitochondrial membrane potential (MMP) that is crucial for driving mitochondrial ATP synthesis. The decline in MMP has been linked to mitochondrial dysfunction that may lead to cell death [29]. Using a cationic fluorescent dye, rhodamine 123, and flow cytometry, we detected a significant reduction in MMP in *Wwox*^+/+^ MEFs after serum starvation in a time-dependent manner (Figure 4C). However, the MMP remained relatively stable in *Wwox*^−/−^ MEFs following serum deprivation (Figure 4C). These results indicate that WWOX triggers MMP loss and increases apoptosis in MEFs in response to a stress condition due to the lack of serum growth factors.

### 3.4. WWOX Downregulates Protein Expression Levels of Bcl-X_L_ and Mcl-1 Under Serum Starvation

**Figure 5:**
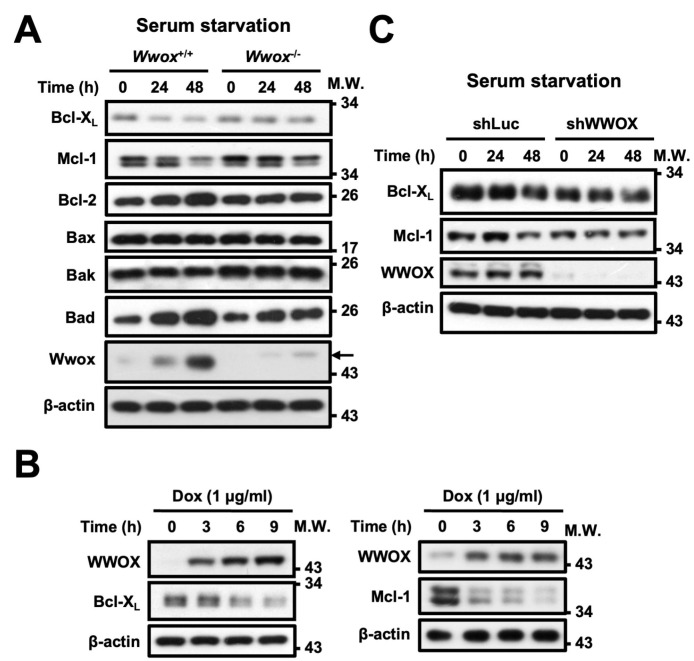
WWOX increases Bcl-X_L_ and Mcl-1 protein downregulation. (**A**) *Wwox*^+/+^ and *Wwox*^−/−^ MEFs were treated with serum-free RPMI-1640 medium for 24 and 48 h. The expression levels of Bcl-2 family members and WWOX protein were determined by Western blotting. The arrow indicates a ~48 kDa non-specific band. (**B**) Ectopic WWOX protein expression was induced by the addition of 1 μg/mL doxycycline (Dox) in HeLa Tet-On cells. After the indicated time intervals, protein levels of WWOX, Bcl-X_L,_ and Mcl-1 were detected by Western blot analysis. (**C**) SCC-15 cells infected with lentivirus expressing a control shRNA (shLuc) or *WWOX* shRNA (shWWOX) were treated with serum-free medium for 24 and 48 h. The protein expression levels were examined by Western blot analysis. β-actin was used as a loading control. The representative results from at least four repeated experiments are shown. Densitometric analysis for quantification of protein levels is shown in Figure S2.

To further explore the mechanism by which WWOX regulates serum starvation-induced cell death, we checked the expression levels of Bcl-2 family proteins that control mitochondrial membrane permeability at the mitochondrial outer membrane for regulating caspase-mediated induction of apoptosis. As shown in Figure 5A, a greater extent of reduction in the expression of anti-apoptotic proteins Bcl-X_L_ and Mcl-1 was detected in *Wwox*^+/+^ MEFs than in *Wwox*^−/−^ MEFs after serum starvation. On the other hand, we observed a marked increase in protein expression of pro-apoptotic Bad in *Wwox*^+/+^ MEFs after serum starvation, and to a lesser extent in *Wwox*^−/−^ MEFs (Figure 5A and Figure S2A). No great changes in the expression of other family member proteins, Bcl-2, Bax, or Bak, were found in these cells following serum starvation (Figure 5A). To further confirm these findings, induction of ectopic WWOX protein expression in HeLa cells by the addition of a tetracycline antibiotic, doxycycline (Dox), using a tetracycline-controlled transcriptional activation (Tet-on) system was tested in this study. Our data showed that Dox-induced ectopic WWOX upregulation was accompanied by a significant reduction in Bcl-X_L_ and Mcl-1 protein expression in HeLa cells (Figure 5B and Figure S2B). Conversely, compared to the downregulation of Bcl-X_L_ and Mcl-1 levels in control cells, lentiviral short hairpin RNA (shRNA)-mediated knockdown of WWOX expression (shWWOX) in SCC-15 cells prevented the reduction in Bcl-X_L_ and Mcl-1 protein expression upon serum starvation (Figure 5C and Figure S2C). These results suggest that upregulation of *Wwox* in cells is associated with Bcl-X_L_ and Mcl-1 downregulation during stress responses.

### 3.5. WWOX Promotes Bcl-X_L_/Mcl-1 Protein Degradation Through a Lysosomal Pathway

**Figure 6:**
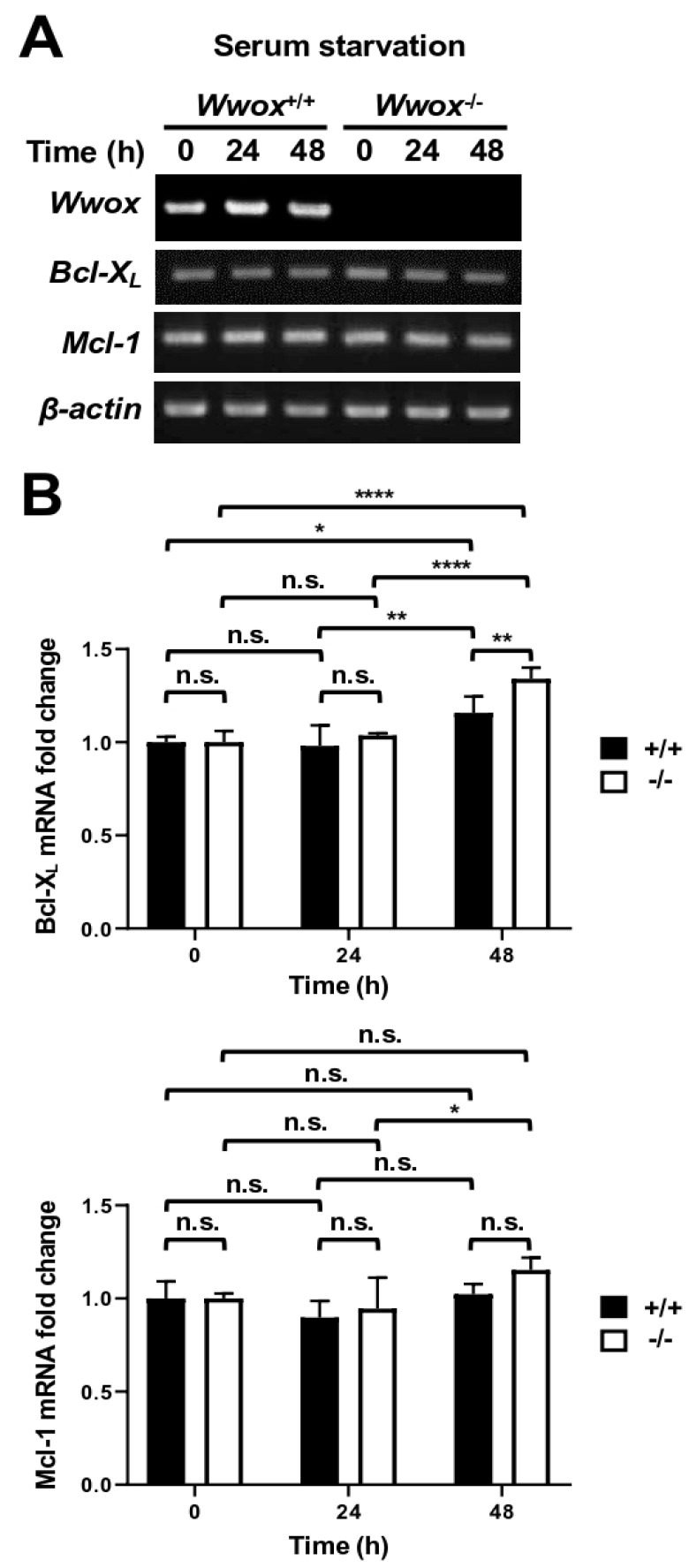
WWOX-mediated Bcl-X_L_/Mcl-1 protein downregulation is not due to a reduction in *Bcl-X_L_* and *Mcl-1* gene transcription. *Wwox*^+/+^ and *Wwox*^−/−^ MEFs were treated with serum-free RPMI-1640 medium. The RNA samples collected at the indicated time points were used to perform reverse transcription to generate cDNA. *Bcl-X_L_* and *Mcl-1* mRNA expression levels in cells were analyzed by PCR followed by agarose gel electrophoresis (**A**) and quantitative real-time PCR (**B**) (n = 4). *β-actin* mRNA levels were used as an internal control. *, *p* ≤ 0.05; **, *p* ≤ 0.01; ****, *p* ≤ 0.001; n.s., not significant.

To investigate whether WWOX decreases Bcl-X_L_ and Mcl-1 expression through transcriptional repression after serum starvation, we analyzed the mRNA levels of *Bcl-X_L_* and *Mcl-1* in MEFs by both traditional RT-PCR (Figure 6A) and real-time quantitative PCR (Figure 6B). We found that the mRNA levels of *Bcl-X_L_* and *Mcl-1* were not decreased after serum starvation in either *Wwox^+/+^* or *Wwox*^−/−^ MEFs (Figure 6), suggesting that downregulation of Bcl-X_L_ and Mcl-1 protein expression by WWOX is not mediated through transcriptional repression.

**Figure 7:**
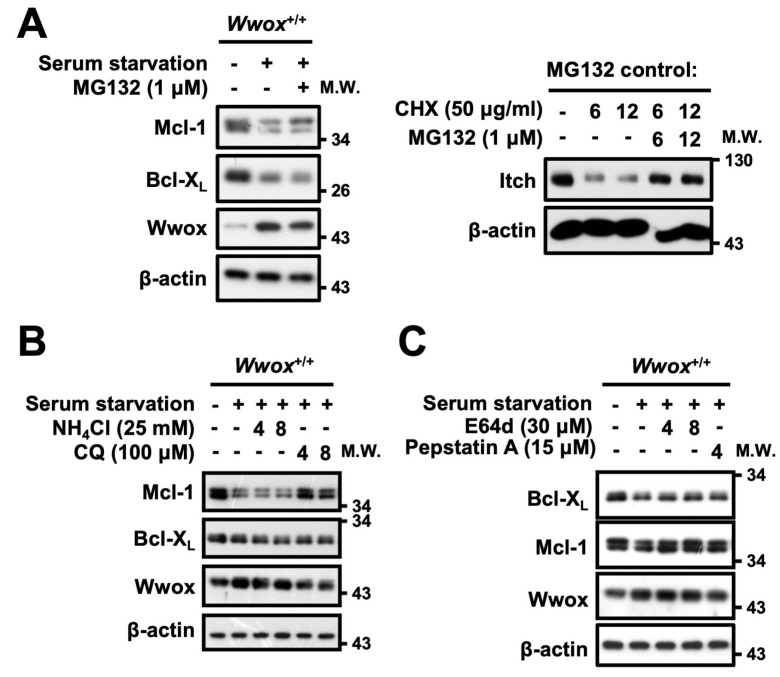
WWOX promotes Bcl-X_L_ and Mcl-1 protein downregulation through a lysosomal degradation pathway. (**A**) *Wwox*^+/+^ MEFs were treated with serum-free medium for 36 h with or without the presence of a proteasome inhibitor, MG132 (1 μM), during the last 18 h of serum starvation. (**B**) *Wwox*^+/+^ MEFs were treated with serum-free medium for 32 h with or without the presence of NH_4_Cl (25 mM) or a lysosome inhibitor, chloroquine (CQ; 100 μM), during the last 4 and 8 h of serum starvation. (**C**) *Wwox*^+/+^ MEFs were treated with serum-free medium for 32 h with or without the presence of a lysosomal enzyme inhibitor E64d (30 μM) or pepstatin A (15 μM) during the last 4 and 8 h of serum starvation. Bcl-X_L_, Mcl-1, and WWOX protein expression was examined by Western blotting. β-actin was used as a loading control. Inhibition of Itch protein degradation in cycloheximide (CHX)-treated cells was used as a MG132 reagent control. The representative results from four repeated experiments are shown. Densitometric analysis for quantification of protein levels is shown in Figure S3.

We further examined whether WWOX decreases Bcl-X_L_ and Mcl-1 protein stability upon stress. The proteasome and lysosome are two major pathways that mediate intracellular protein degradation. To study whether WWOX facilitates Bcl-X_L_ and Mcl-1 protein degradation via the proteasomal pathway, a proteasome inhibitor, MG132, was used to block proteasome-mediated protein degradation upon serum starvation (Figure 7A). Our results revealed that MG132 treatment failed to prevent protein downregulation of Bcl-X_L_ and Mcl-1 in *Wwox*^+/+^ MEFs upon serum starvation (Figure 7A and Figure S3A). Interestingly, treatment of *Wwox*^+/+^ MEFs with a lysosome inhibitor, chloroquine (CQ), blocked serum starvation-induced Mcl-1 protein reduction (Figure 7B and Figure S3B). Autophagy is a highly regulated cellular process that can lead to bulk protein degradation or the removal of damaged organelles and regenerate free amino acids and fatty acids required for maintaining energy levels and essential cellular functions to endure a hostile environment, including nutrient deprivation or stress responses [30]. Membrane-permeable E64d and pepstatin A are commonly used as two autophagy inhibitors that can directly bind to and block the activities of cysteine and aspartic proteases, respectively, in autophagolysosomes [31]. The presence of E64d and pepstatin A reversed Bcl-X_L_ and Mcl-1 protein downregulation after serum starvation in *Wwox*^+/+^ MEFs (Figure 7C and Figure S3C). These results suggest that WWOX promotes Bcl-X_L_ and Mcl-1 protein degradation upon serum starvation through the lysosome/autophagy-mediated pathway. Increase mitochondrial targeting of WWOX upon stress enhances cell apoptosis [28]. Co-immunoprecipitation experiments revealed that neither Bcl-X_L_ nor Mcl-1 physically interacted with WWOX. Whether WWOX regulates activation of E3 ligases to facilitate mitochondrial Bcl-X_L_ and Mcl-1 protein degradation for inducing MMP reduction and apoptosis remains to be clarified.

### 3.6. Enhanced Oxidative Stress in Cells Increases WWOX Expression and Downregulates Bcl-X_L_ Under Serum Starvation

**Figure 8:**
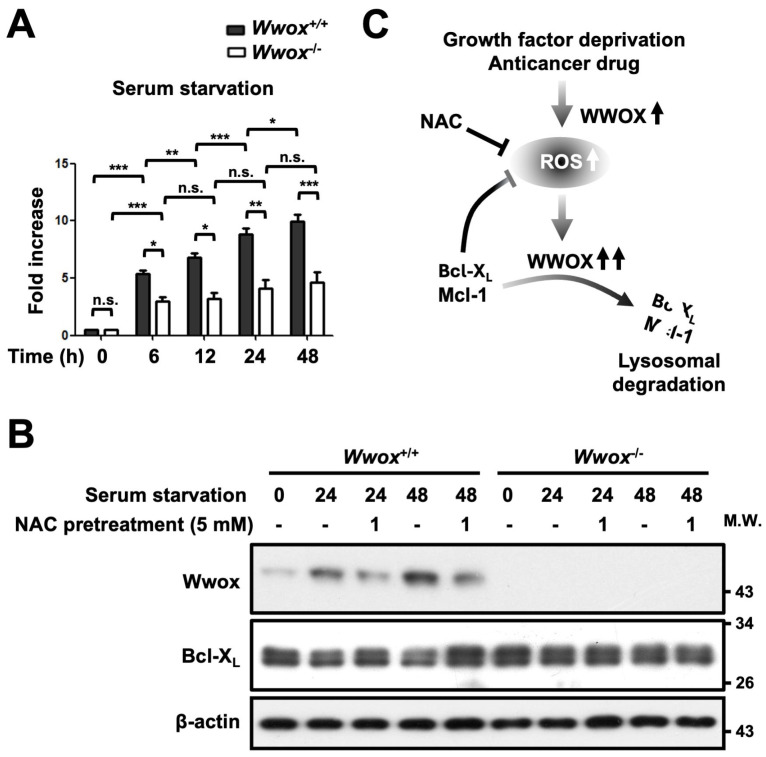
WWOX upregulation enhances intracellular ROS levels upon serum starvation for triggering Bcl-X_L_ protein downregulation. (**A**) *Wwox*^+/+^ and *Wwox*^−/−^ MEFs were treated with serum-free medium for the indicated periods, and ROS levels in cells were detected using DCFH-DA and a flow cytometer. * *p* value < 0.05; ** *p* value < 0.005; *** *p* value < 0.0001; n.s., not significant. (**B**) *Wwox*^+/+^ and *Wwox*^−/−^ MEFs were pretreated with 5 mM NAC for 1 h and then treated with serum-free medium. Total cell lysates were harvested after 24 and 48 h, and protein expression levels of WWOX and Bcl-X_L_ were detected by Western blot analysis. β-actin was used as a loading control. The representative results from four repeated experiments are shown. Densitometric analysis for quantification of protein levels is shown in Figure S4. (**C**) Schematic description of WWOX induction in regulating stress responses.

Because WWOX has been shown to function as a stress-responsive regulator of cellular apoptosis [24,32,33], we further delineated the underlying mechanism that confers WWOX induction upon the stress response during serum starvation. Previous studies have reported that serum starvation triggers the generation of ROS in cells [34,35,36]. Elevated ROS formation, denoted as oxidative stress, leads to oxidative damage in the molecules or organelles of a cell. Using a fluorescent redox probe, dichloro-dihydro-fluorescein diacetate (DCF-DA), we detected increased intracellular ROS levels in both *Wwox*^+/+^ and *Wwox*^−/−^ MEFs after serum starvation in a time-dependent manner (Figure 8A). No differences in the amounts of ROS generation were observed between the freshly isolated *Wwox*^+/+^ and *Wwox*^−/−^ MEFs (Figure 8A). Interestingly, pretreatment of *Wwox*^+/+^ MEFs with an antioxidant NAC for 1 h blocked WWOX upregulation induced by serum starvation, thereby preventing Bcl-X_L_ downregulation (Figure 8B and Figure S4). With the presence of serum, NAC treatment did not change the basal level of WWOX. Taken together, these results suggest that serum starvation-induced oxidative stress increases WWOX expression for triggering lysosomal degradation of Bcl-X_L_ and Mcl-1 proteins in cells, thereby attenuating their protective effect on mitochondrial damage and oxidative stress (Figure 8C) [37,38]. Blockade of ROS production inhibits WWOX induction upon growth factor deprivation, suggesting a reciprocal suppression between WWOX and anti-apoptotic Bcl-2 family proteins (Figure 8C).

## 4. Discussion

The activation of WWOX protein under stress stimuli has been examined in different types of cells [39]. Previous studies have shown that treatment of cells with complement C1q or anticancer drugs may induce protein phosphorylation of WWOX for promoting cancer cell apoptosis [20,40,41]. Stimulation of cells with a commonly used protein kinase inhibitor, staurosporine, a topoisomerase II inhibitor, etoposide, an antimetabolite methotrexate, or tumor necrosis factor activates WWOX protein via triggering its phosphorylation at tyrosine 33 and translocation to the nucleus or mitochondria for inducing apoptosis [20,24,28]. Steroid hormones, such as estrogen and androgen, also induce WWOX activation and nuclear translocation in human breast and prostate cancer cells [27]. Although hyaluronidase treatment has been reported to increase WWOX protein expression in L929 fibroblasts [19], the scenario for the induction of WWOX protein expression remains largely unclear. In this study, we demonstrated that serum starvation-induced oxidative stress triggered WWOX upregulation, thereby leading to a gradual decline in protein levels of Bcl-X_L_ and Mcl-1 through a lysosome/autophagy-mediated pathway and induction of cell apoptosis. Deprivation of serum growth factors or nutrients in the tumor microenvironment induces stress responses that may cause a reduction in cancer cell growth and proliferation, and an increase in apoptosis.

WWOX has been shown to play crucial roles in regulating DNA repair machinery and maintaining genome integrity [21,23,26,42,43]. Mutations or absent expression of WWOX may contribute to cancer progression. Previous studies have shown that progression of breast cancer and skin squamous cell carcinoma to a pre-metastatic stage positively correlates with increased expression of tyrosine 33-phosphorylated WWOX protein [24,27]. Whether precancerous changes in hyperplastic tissues trigger a stress response, which in turn induces WWOX upregulation, is unclear. Significant downregulation of WWOX protein expression in human SCC with poor differentiation or metastatic cancer cells is associated with genome instability and cancer deterioration [24,26,27]. Moreover, WWOX protein expression is significantly decreased in the hippocampal neurons of patients with Alzheimer’s disease [11]. WWOX has been shown to block Tau hyperphosphorylation and neurofibrillary tangle formation for preventing neurodegeneration [44]. *Wwox* deficiency in mice causes neurodevelopmental and degenerative neuropathies, GSK-3β-mediated epileptic seizure, and high embryonic or postnatal lethality [10]. Early lethal microcephaly associated with epileptic seizures, cerebellar ataxia, intellectual disability, and profound developmental delay in pediatric patients with an autosomal recessive mutation of *WWOX* has been linked to a WOREE syndrome [45,46,47]. Thus, the understanding of WWOX expression control is important for developing therapeutic strategies for human malignancies and neuronal diseases.

Ultraviolet (UV) irradiation also induces oxidative stress and increases protein expression and tyrosine 33 phosphorylation of WWOX in cells [24,48]. The exposure of cells to low-dose UV irradiation causes DNA damage, induces cell cycle arrest, and triggers WWOX activation and signaling pathways for DNA repair to maintain genome integrity. As the damages are beyond repair after treatment of cells with high-dose UV irradiation, a large extent of WWOX upregulation and stress protein activation may induce apoptosis for culling the severely damaged cells. Treatment of cells with the commonly used anticancer drug etoposide or cisplatin may cause DNA damage through targeting topoisomerase II activity or inducing DNA crosslinking, respectively. These antineoplastic agents have been shown to generate DNA breaks and trigger apoptosis via inducing ROS accumulation within cancer cells [49]. WWOX upregulation induced by oxidative stress may contribute to the induction of apoptosis in anticancer drug-treated cells.

We determined increased transcription of the *WWOX* gene in both cell lines and primary fibroblasts after treating the cells with various stress stimuli, including serum deprivation and H_2_O_2_. However, how the stress response triggers transcriptional activation of the *WWOX* gene and what factors are involved in activating the *WWOX* promoter are largely unclear. WWOX is crucial for mediating cancer suppression, bone metabolism, cell differentiation, and embryonic development [10,12,24,50,51]. Therefore, delineating the regulation of the *WWOX* gene transcription is important for establishing therapeutic strategies for patients with disorders associated with dysregulation of WWOX expression. CpG island hypermethylation of the *WWOX* promoter sequence silences gene transcription in human lung cancer cells [52]. The ring finger protein Bmi1 is a major component of polycomb group complex 1 that functions as an epigenetic repressor of many genes involved in embryonic development, stem cell self-renewal, oxidative stress, and DNA repair pathways [53,54,55]. Bmi1 suppresses WWOX expression through directly binding to the *WWOX* promoter and plays an oncogenic role in small cell lung cancer [56].

Oncogenic microRNA has been reported to modulate WWOX expression in cancer cells [57]. MicroRNA 134 (miR-134) targets the 3′ untranslated region of *WWOX* transcript and represses WWOX expression in head and neck squamous cell carcinoma for promoting cancer progression [57]. Whether stress responses increase WWOX protein expression through modulating microRNA levels (e.g., downregulation of miR-134) remains to be studied. Tyrosine kinase Ack1 phosphorylates WWOX protein at tyrosine 287 to facilitate WWOX polyubiquitination and degradation through a proteasomal pathway [58]. However, no significant changes in WWOX protein phosphorylation at tyrosine 287 were detected in MEFs after serum starvation, suggesting that WWOX upregulation induced by serum growth factor deprivation may not be mediated via inhibiting Ack1.

We determined that WWOX upregulation decreases Bcl-X_L_ and Mcl-1 protein levels in cells upon serum starvation through a lysosomal degradation pathway but not transcriptional repression. However, the possibility still exists that WWOX may downregulate Bcl-X_L_ and Mcl-1 protein translation. Interestingly, Bcl-X_L_ mRNA contains an internal ribosome entry site (IRES) within its 5′ untranslated region (5′UTR) for recruiting RNA-interacting factors that may initiate cap-independent translation [59]. Many genes containing an IRES sequence at 5′UTR encode proteins that may regulate cell proliferation, survival, and apoptosis [60,61]. It has been shown that IRES-mediated translation still occurs under stress conditions such as serum starvation and irradiation exposure, as cap-dependent protein synthesis is inhibited in cells [62,63]. Whether WWOX participates in suppressing IRES-mediated translation of Bcl-X_L_ protein during serum starvation is unclear.

A previous study has indicated that Mcl-1 protein is highly unstable and can be rapidly ubiquitinated by an E3 ligase MULE/ARF-BP1 for degradation through a proteasome-dependent pathway [64]. We found in this study that WWOX downregulates Mcl-1 protein expression in cells via a lysosomal degradation pathway during serum starvation. These results suggest that the processes of Mcl-1 protein degradation can be operated through two distinct mechanisms under different conditions. Treatment of cells with CQ inhibits autophagosome fusion with lysosomes and prevents Mcl-1 protein degradation through a macroautophagic pathway upon serum starvation. Lysosomal protease inhibitors E64d and pepstatin A block the proteolysis of cargo proteins in autophagolysosomes. Because pharmacological inhibitors may have pleiotropic effects, knockdown of essential genes involved in the autophagy or lysosomal degradation pathway using RNA interference tools needs to be tested for verification.

We found that the protein expression levels of proapoptotic Bax and Bak remained unchanged following serum starvation in both wild-type and *Wwox* knockout MEFs. Downregulation of antiapoptotic Bcl-XL and Mcl-1 proteins may induce oligomerization of Bax and Bak at the mitochondrial outer membrane, thus leading to the release of cytochrome c and other proapoptotic factors for triggering cell death. Our results suggested that WWOX-mediated Bcl-XL and Mcl-1 degradation could be the initiating and a pivotal event in the induction of mitochondrial apoptosis.

Starvation may trigger ROS accumulation and stress-related signaling pathways such as JNK signaling in cells [65,66]. WWOX is associated with the metabolic transition of glycolysis to mitochondrial oxidative phosphorylation and redox homeostasis in cells [13,26,51]. In addition, WWOX regulates the expression of enzymes related to aerobic metabolism and ROS generation in *Drosophila* [67]. Serum starvation and oxidative stress induce WWOX upregulation that positively correlates with increased ROS levels in cells. Serum starvation may upregulate WWOX or other enzymes with oxidoreductase activity, such as NADPH oxidase, to promote ROS generation [68]. Excessive production of ROS in cells after serum starvation may further increase WWOX expression through ignition of oxidative stress-induced signal transduction. Moreover, WWOX upregulation may promote protein degradation of Bcl-2 family members Bcl-X_L_ and Mcl-1 and affect mitochondrial redox homeostasis. These findings suggest that WWOX may participate in a feedback loop of serum starvation-induced ROS production (Figure 8C). Intriguingly, *Wwox* knockout MEFs maintained in complete medium containing 10% FBS generate higher ROS levels as compared with the wild-type controls after in vitro culture for ~30 passages [26]. Whether WWOX is involved in the initiation of signal induction for serum starvation-triggered ROS production and ROS downstream signaling needs further delineation.

## 5. Conclusions

The loss or mutations of WWOX may cause many human diseases, including cancers, Alzheimer’s disease, epileptic seizures, growth retardation, and early postnatal lethality. Although extensive efforts have been made to elucidate the functional roles of WWOX in controlling cell growth, differentiation, and cancer development, the regulation of WWOX induction in cells needs to be clarified. Our results provide clear evidence that environmental stress conditions, including growth factor deprivation and chemotherapy, increase WWOX protein expression through transcriptional induction in cells. The observed effects of WWOX induction in cells highlight its contribution to deficits in mitochondrial membrane potential and increased cell death under stress. WWOX upregulation triggers lysosomal degradation of Bcl-X_L_ and Mcl-1 proteins, thereby attenuating their protective effect on mitochondrial damage and oxidative stress. Blockade of ROS inhibits WWOX induction upon growth factor deprivation, suggesting the reciprocal suppression between WWOX and anti-apoptotic Bcl-2 family proteins Bcl-X_L_ and Mcl-1 (Figure 8C). Conceivably, delineation of the molecular mechanism by which stress stimulation increases WWOX for regulating cellular functions will have a great impact on our understanding of many human diseases. WWOX is apparently an attractive target for therapeutic intervention. These findings raise important translational implications for treating patients with disorders due to WWOX deficiency.

## Figures and Tables

**Table 1:** Sequences of PCR primers and lentiviral shRNA.

PCR Primers		
Human *WWOX*	Forward 5′-AAAACGACTATTGGGCGATG Reverse 5′-GTGTTGGAGGGACATTTGGA	PCR product: 491 bp
Human *β-actin*	Forward 5′-AGCGGGAAATCGTGCGTG Reverse 5′-CAGGGTACATGGTGGTG	PCR product: 309 bp
Mouse *Wwox*	Forward 5′-ACTACGCCAATCACACTGAGG Reverse 5′-GTCCACGGTAAATGCCAATC	PCR product: 188 bp
Mouse *β-actin*	Forward 5′-TGGAATCCTGTGGCATCCATGAAAC Reverse 5′-TAAAACGCAGCTCAGTAACAGTCCG	PCR product: 349 bp
Mouse *Bcl-X_L_*	Forward 5′-CGGAGAGCGTTCAGTGATCTA Reverse 5′-CGACTCACCAATACCTGCATC	PCR product: 191 bp
Mouse *Mcl-1*	Forward 5′-CTCTTAAAGCTCCAGCCACCA Reverse 5′-GCCACAATCCTGTAGCCACT	PCR product: 143 bp
**Lentiviral shRNA**		
Human sh*WWOX*	5′-CCGGGCCAAGAATGTGCCTCTTCATCTCGAGATGAAGAGGC ACATTCTTGGCTTTTTG	
sh*Luc*	5′-CCGGGCGGTTGCCAAGAGGTTCCATCTCGAGATGGAACCTC TTGGCAACCGCTTTTTG	

## Data Availability

The data that support the findings of this study are available from the corresponding author upon reasonable request.

## Supplementary Materials

The following supporting information can be downloaded at: https://www.mdpi.com/article/10.3390/cells15030270/s1, Figure S1. Treatment with anticancer drugs increases WWOX protein expression in SCC-15 cells. Figure S2. WWOX regulates Bcl-2 family protein expression. Figure S3. WWOX enhances Bcl-xL and Mcl-1 protein degradation via a lysosomal degradation pathway. Figure S4. NAC treatment inhibits WWOX upregulation and Bcl-XL protein downregulation upon serum starvation in wild-type MEFs.
