# Supplementary material for: WWOX Induction Promotes Bcl-XL and Mcl-1 Degradation Through a Lysosomal Pathway upon Stress Responses
Source: Cells. 2026 Jan 31;15(3):270. doi: 10.3390/cells15030270 (PMC12897155; doi:10.3390/cells15030270)
Supplement: Supplementary file 1 [file cells-15-00270-s001.zip › cells-4078288-supplementary.pdf]

WWOX induction promotes Bcl-X_L and Mcl-1 degradation through a lysosomal pathway upon stress responses

Yu-Han Su[1,#], Wei Chiang[2,#], Yi-Yu Wang[1], Yi-Hsi Kung[1], Pai-Shan Cheng[3,*], Tsung-Hao Chang[2], Nan-Shan Chang[4], Feng-Jie Lai[3,5,*], Li-Jin Hsu[1,2,6,7,*]

[1]Department of Medical Laboratory Science and Biotechnology, College of Medicine, National Cheng Kung University, Tainan 701401, Taiwan

[2]Institute of Basic Medical Sciences, College of Medicine, National Cheng Kung University, Tainan 701401, Taiwan

[3]Department of Dermatology, Chi Mei Medical Center, Tainan 710402, Taiwan

[4]Graduate Institute of Biomedical Sciences, China Medical University, Taichung 404328, Taiwan

[5]Center for General Education, Southern Taiwan University of Science and Technology, Tainan 710301, Taiwan

[6]Center of Infectious Disease and Signaling Research, College of Medicine, National Cheng Kung University, Tainan 701401, Taiwan

[7]Research Center for Medical Laboratory Biotechnology, College of Medicine, National Cheng Kung University, Tainan 701401, Taiwan

[#] These authors contributed equally.

[*] Address correspondence to:

L.-J. Hsu, Department of Medical Laboratory Science and Biotechnology, College of Medicine, National Cheng Kung University, Tainan 701401, Taiwan.

Email: ljhsu@mail.ncku.edu.tw; hsu.lijin@gmail.com

F.-J. Lai, Department of Dermatology, Chi Mei Medical Center, Tainan 710402, Taiwan.

Email: lai.fengjie@gmail.com

P.-S. Cheng, Department of Dermatology, Chi Mei Medical Center, Tainan 710402, Taiwan.

Email: coshan927@hotmail.com

Supplementary Figure S1

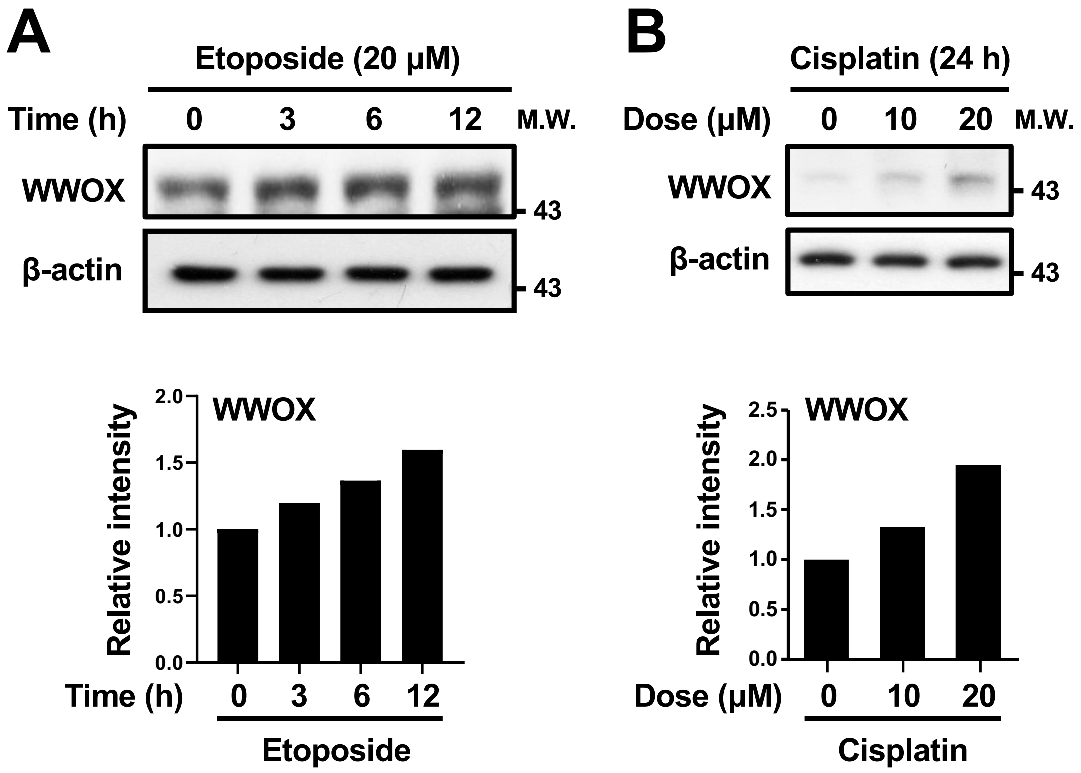

Figure S1. Treatment of anticancer drugs increases WWOX protein expression in SCC-15 cells. WWOX protein levels in SCC-15 cells treated with anticancer drugs etoposide (A) and cisplatin (B) for the indicated time intervals were determined by western blot analysis. β-actin was used as a loading control. The lower panel shows densitometric analysis. M.W., molecular weight (kDa).

Supplementary Figure S2

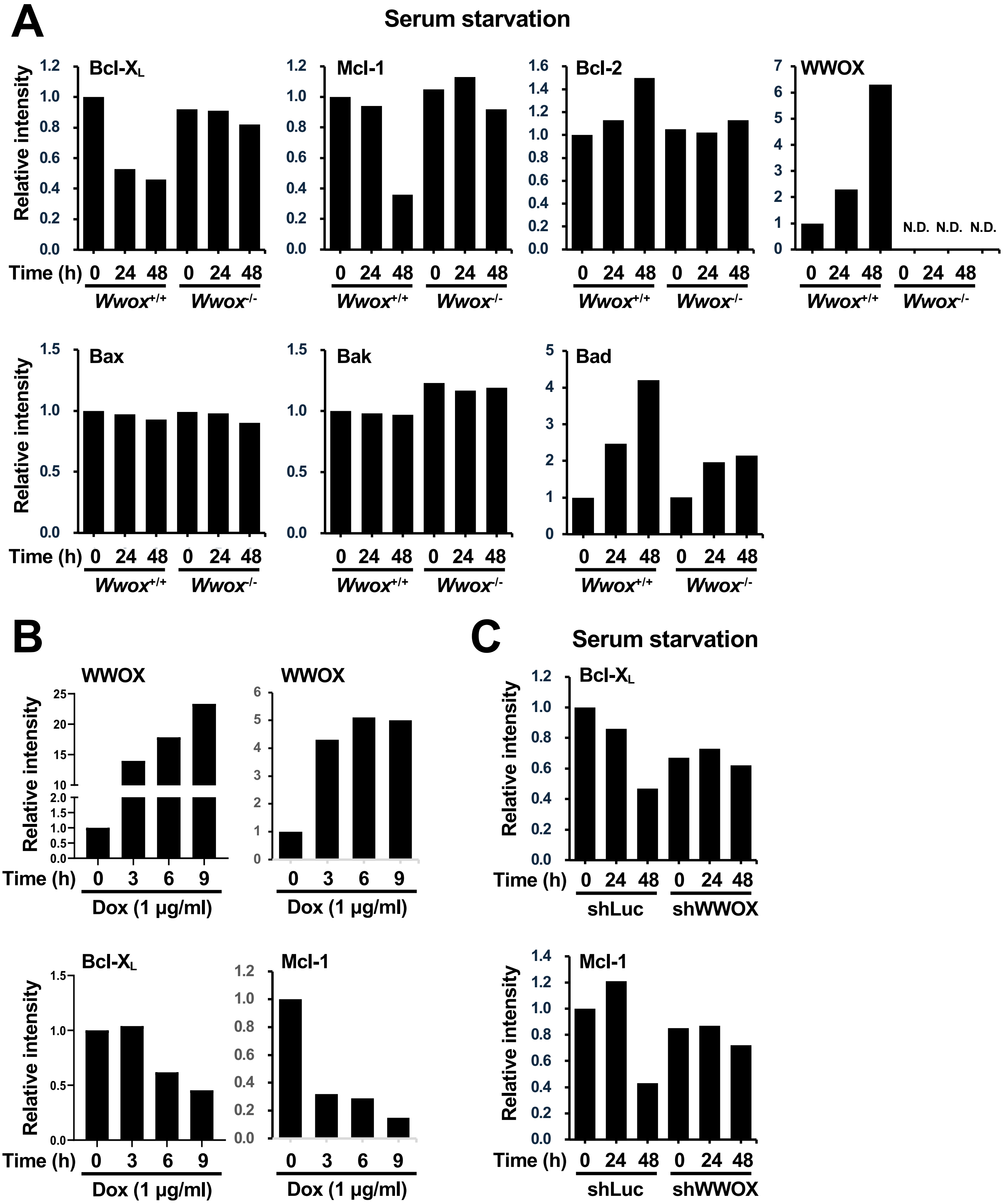

Figure S2. WWOX regulates Bcl-2 family protein expression. (A-C) Densitometric analysis of the data shown in Fig. 5A-C using β-actin as an internal control for quantification of protein expression levels.

Supplementary Figure S3

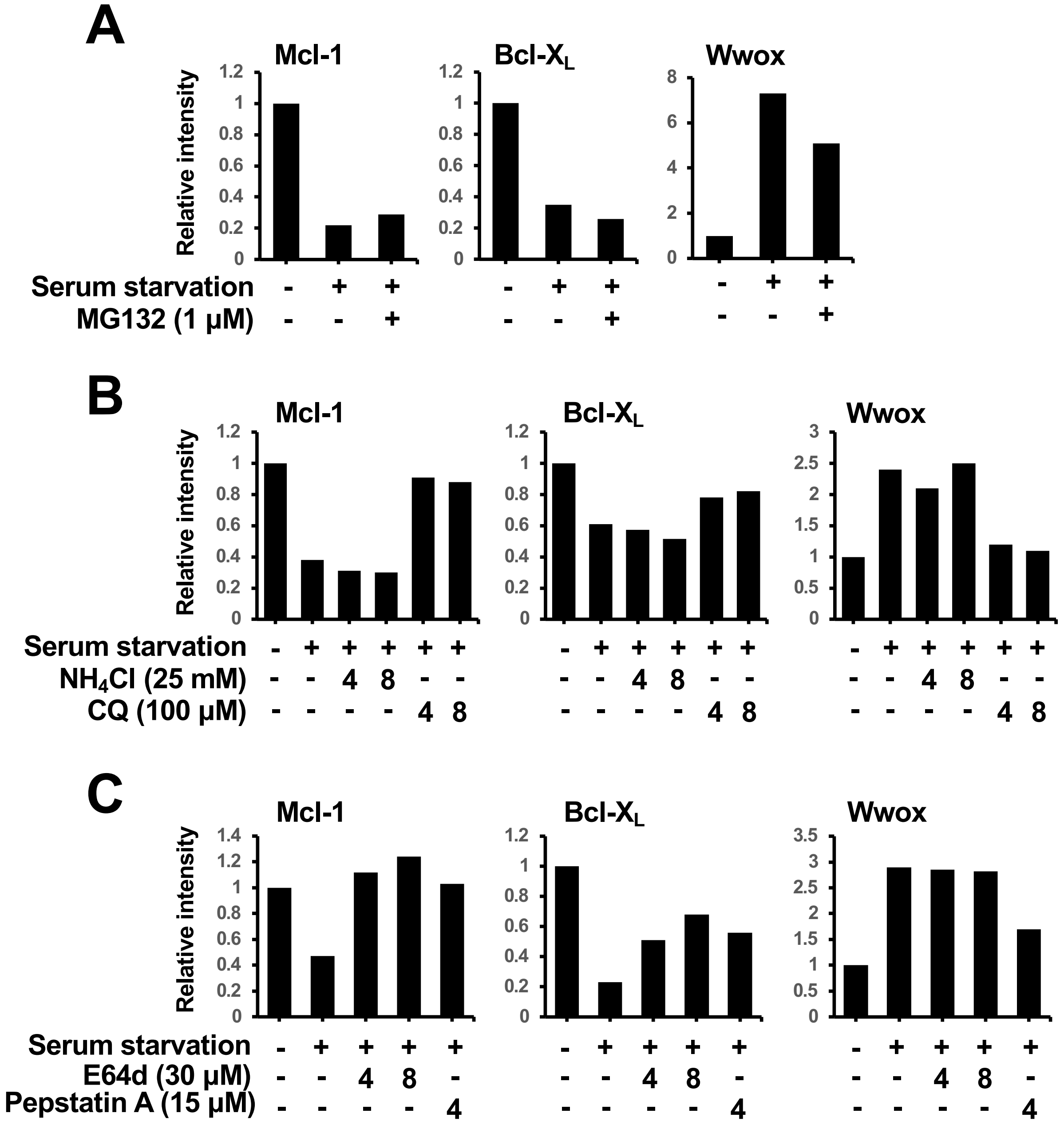

Figure S3. WWOX enhances Bcl-xL and Mcl-1 protein degradation via a lysosomal degradation pathway. (A-C) Densitometric analysis of the data shown in Fig. 7A-C using β-actin as an internal control for quantification of protein expression levels.

Supplementary Figure S4

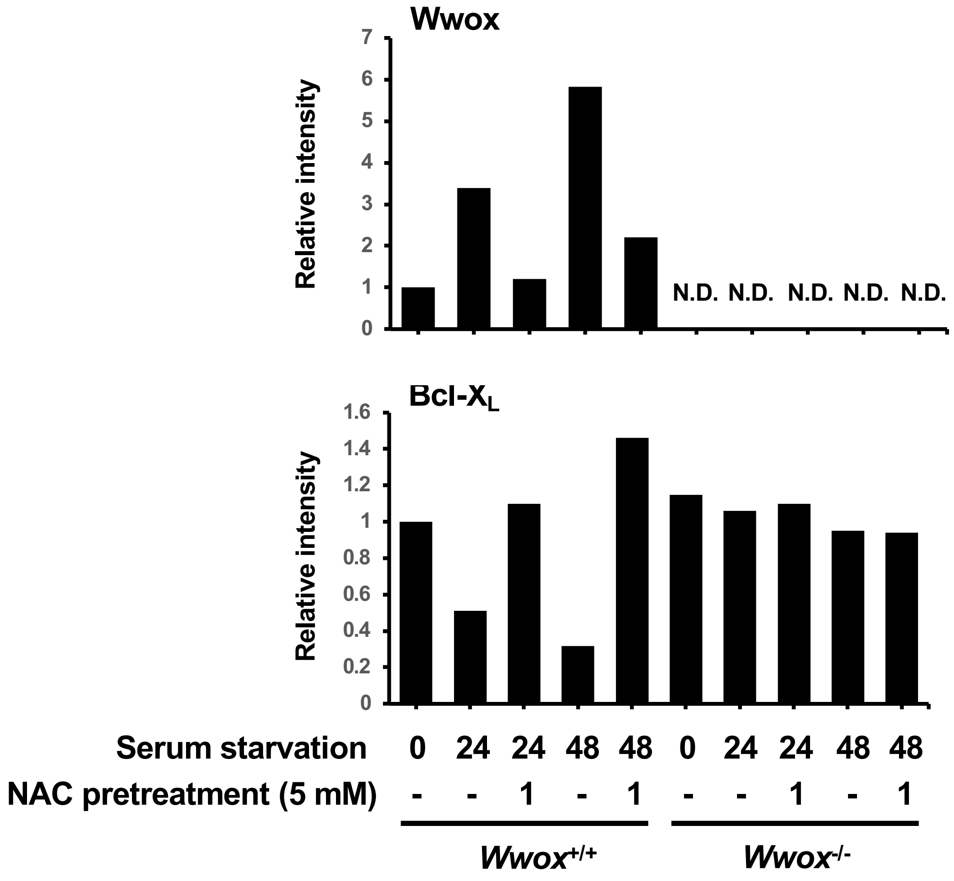

Figure S4. NAC treatment inhibits WWOX upregulation and Bcl-X_L protein downregulation upon serum starvation in wild-type MEFs. Densitometric analysis of the data shown in Fig. 8B using β-actin as an internal control for quantification of protein expression levels.
